# Auditory steady state responses and cochlear implants: Modeling the artifact-response mixture in the perspective of denoising

**DOI:** 10.1371/journal.pone.0174462

**Published:** 2017-03-28

**Authors:** Faten Mina, Virginie Attina, Yvan Duroc, Evelyne Veuillet, Eric Truy, Hung Thai-Van

**Affiliations:** 1 Claude Bernard Lyon 1 University, Lyon, France; 2 Lyon Neuroscience Research Center (Inserm U1028 CNRS UMR5292), Lyon, France; 3 Department of Audiology and Otoneurological Evaluation, Civil Hospitals of Lyon, Lyon, France; 4 ENT Department, Civil Hospitals of Lyon, Lyon, France; Harvard Medical School, UNITED STATES

## Abstract

Auditory steady state responses (ASSRs) in cochlear implant (CI) patients are contaminated by the spread of a continuous CI electrical stimulation artifact. The aim of this work was to model the electrophysiological mixture of the CI artifact and the corresponding evoked potentials on scalp electrodes in order to evaluate the performance of denoising algorithms in eliminating the CI artifact in a controlled environment. The basis of the proposed computational framework is a neural mass model representing the nodes of the auditory pathways. Six main contributors to auditory evoked potentials from the cochlear level and up to the auditory cortex were taken into consideration. The simulated dynamics were then projected into a 3-layer realistic head model. 32-channel scalp recordings of the CI artifact-response were then generated by solving the electromagnetic forward problem. As an application, the framework’s simulated 32-channel datasets were used to compare the performance of 4 commonly used Independent Component Analysis (ICA) algorithms: *infomax*, *extended infomax*, *jade* and *fastICA* in eliminating the CI artifact. As expected, two major components were detectable in the simulated datasets, a low frequency component at the modulation frequency and a pulsatile high frequency component related to the stimulation frequency. The first can be attributed to the phase-locked ASSR and the second to the stimulation artifact. Among the ICA algorithms tested, simulations showed that *infomax* was the most efficient and reliable in denoising the CI artifact-response mixture. Denoising algorithms can induce undesirable deformation of the signal of interest in real CI patient recordings. The proposed framework is a valuable tool for evaluating these algorithms in a controllable environment ahead of experimental or clinical applications.

## 1 Introduction

The ongoing development of cochlear implants (CIs) has continuously improved the quality of life of patients suffering from profound hearing loss by compensating the dysfunction of the cochlea’s sensory hair cells with adequate stimulation currents. In practice, the CI stimulation parameters must be adjusted patient-wise in consideration of the subjective evolution of auditory perception after cochlear implantation. This is performed during regular post-implantation CI parameter fitting sessions. During each session, a subjective evaluation of the threshold and comfortable hearing levels is determined for each CI stimulation electrode (up to 22 electrodes depending on the manufacturer).

While the active participation of adult patients in these time-consuming fitting sessions is indispensable, behavioral indices constitute the only guide for the adjustment of CI parameters in infants, toddlers, the disabled and the elderly. Consequently, and in view of the absence of clear clinical guidelines for current CI parameter fitting practices [1], objective measures of auditory perception in cochlear implanted patients have long been a subject of interest for the hearing community [2–9]. These measures represent a solution for optimizing CI parameters without requiring the active participation of the patient.

Nevertheless, state-of-the-art studies [10–13] have highlighted the complexity of detecting these objective measures, mainly evoked potentials, in the presence of the electrical artifact that is generated by the CI as well as the difficulty in suppressing this artifact. Therefore, the reliability of these responses remains, in some cases, bound to the subjective judgment and experience of the clinician or the experimenter. For instance, responses may be considered valid based on their scalp topographical distribution [14] or other electrophysiological assumptions such as the amplitude growth function [12]. These assumptions are usually based on equivalent results obtained through experiments with normal hearing subjects [15, 16].

Among the different types of objective measures, auditory steady state responses (ASSRs) are of particular interest given the reported correlation of their amplitude growth function with the loudness growth function of auditory stimulus in normal hearing subjects [15]. More importantly, recent studies demonstrated the reliability and feasibility of using ASSRs in the context of CI clinical fittings [11, 17–19]. ASSRs can also be used for testing multiple hearing frequencies simultaneously and are thus less time-consuming than other conventional auditory evoked potentials (see [16] for a review).

To our knowledge, the interference of the CI stimulation artifact with the detection of the evoked responses and therefore the reliability of objective measures in CI patients has never been formally addressed. This is probably due to the fact that this is practically impossible to achieve in clinical data given the physical co-existence of the artifactual activation source, the CI, and the electrophysiological sources of these evoked potentials in CI patients. It is generally assumed that a significant difference between the phase value of the detected response and that of the corresponding contaminated raw data is sufficient proof of reliable detection. The objective of the present study is to propose a formal approach for: 1) evaluating the efficiency of commonly used signal processing algorithms in suppressing the CI artifact from EEG datasets and 2) assessing their impact on the characteristics of the evoked electrophysiological responses.

Blanking [19] is one of the most commonly reported denoising algorithms used to eliminate the CI artifact in the context of ASSR. This method is particularly efficient in eliminating symmetric stimulation pulses of short duration and has a proven reliability in denoising ASSR clinical data ahead of response detection [11, 18, 19]. Nevertheless, the performance of this algorithm remains bound to the limited temporal spread of the CI artifact. In fact, in a very recent clinical study, the authors report that blanking was insufficient for the suppression of electrical stimulation artifacts from the electrophysiological recordings of ipsilateral electrodes [18]. Although ASSRs were retrievable from less contaminated contralateral electrodes, the mentioned study concludes by acknowledging the need for the development of a “more sophisticated” denoising method capable of removing the artifact from all recording electrodes. Moreover, the stimulation paradigm of cochlear implants varies among manufacturers which can make it difficult for the blanking method to function universally. For instance, the Digisonic SP cochlear implant (Oticon Medical, Vallauris, France) employs pulse-width-modulation to encode sound intensity. This implant generates pseudo monophasic pulse trains in order to maximize the probability of exciting the auditory nerve fibers [20]. Each monophasic pulse then resembles an active anodic-first pulse followed by an asymmetric passive discharge that counterbalances the accumulation of electrochemical Faraday charges. This passive discharge is exponential in shape and of relatively long-term duration, i.e. the contamination induced by each pulse can extend over 1 ms, equivalent to 50% of the inter-active-pulse interval at a clinical stimulation rate of 500 Hz. Therefore, using the standard blanking method, in this context, results in the elimination of a large portion of the useful signal. Consequently, the use of an alternative denoising method such as independent component analysis (ICA) may be more suitable to eliminate such temporally-spread electrical artifacts. A recent study reported that ICA (*infomax*) was more efficient than blanking in denoising the CI artifact in clinical data and therefore retrieving less contaminated ASSR measures [17].

In this paper, a computational simulation framework is proposed. It aims to simulate the mixture of the CI artifact and the corresponding electrically evoked responses. Its parameters were tuned to represent the Digisonic SP cochlear implant’s stimulation paradigm. This computational framework is built on a neural mass model of the main generators of auditory evoked potentials (AEPs) as well as the stimulation source, the CI. A realistic head model was then used in order to simulate the 10–10 EEG datasets, which were used to test the performance of candidate signal processing algorithms in separating the artifact-response mixture. Four ICA algorithms were explored in simulation. Finally, an illustration of the effects of these methods on the detected responses in a CI patient was also performed.

## 2 Materials and methods

A formal approach for quantifying the efficiency of a signal processing algorithm in suppressing the CI stimulation artifact from the EEG recordings of a CI patient consists in providing access to the expected uncontaminated recordings. Since this is impossible to achieve in real clinical datasets, we propose a computational framework allowing for the separate simulation of 1) the artifactual CI electrical potentials and 2) the corresponding evoked electrophysiological potentials. This section presents the framework’s architecture, from the level of the neural mass model to the realistic head model, as well as the methodology used to evaluate and compare the performance of four ICA algorithms in denoising the artifact-response mixture. Finally, the clinical dataset used for the preliminary exploration of the outcome of these algorithms in real data is described.

### 2.1 The lumped-parameter model

**Fig 1:**
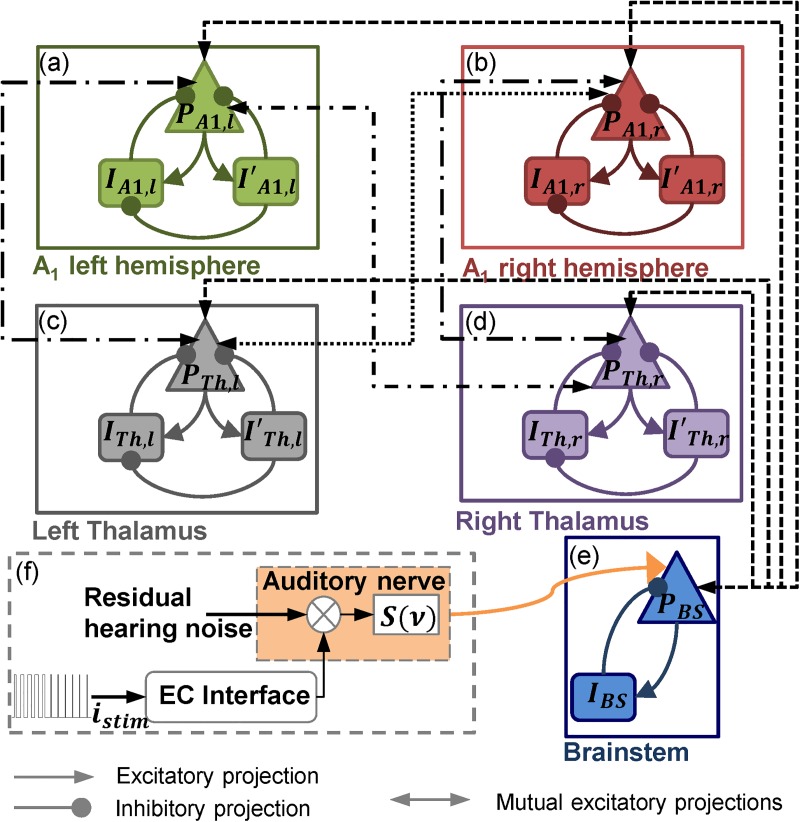
Architecture of the neural mass model: (a) left cortical module, (b) right cortical module, (c) left thalamic module, (d) right thalamic module, (e) brainstem module and (f) cochlear module.

In order to simulate realistic neural dynamics, the proposed computational framework was based on a lumped-parameter model of auditory neural dynamics. This model was built on the mesoscopic level for two reasons. First, the main objective of this framework is to evaluate the efficiency of artifact removal methodologies, i.e. artifact-response separability. Therefore, a microscopic representation of the underlying neural dynamics of auditory processing was considered superfluous. Second, at this level, we were not interested in the effectiveness of auditory evoked potential (AEP) detection algorithms. Consequently, the macroscopic modeling of the background cortical activity constituted an unnecessary additional computational complexity for this computational framework. However, in conformity with the neurophysiological generation of ASSRs, the representation of the main neural generators [21] was judged indispensable. Therefore, the proposed lumped-parameter model included six interacting modules: two cortical modules, two thalamic modules, a brainstem module and a cochlear module (see Fig 1).

#### 2.1.1 The cortical modules

The cortical modules represent the two processing nodes of the left (Fig 1A) and the right primary auditory cortex (Fig 1B) denoted *A*_*1*,*l*_ and *A*_*1*,*r*_ respectively. Besides a principal cell neuronal population, (*P*_*A1*,*l*_ and *P*_*A1*,*r*_), these modules include two interneuron populations representing fast-mediated (*I*_*A1*,*l*_ and *I*_*A1*,*r*_) and slow-mediated GABAergic transmission (*I’*_*A1*,*l*_ and *I’*_*A1*,*r*_). The output of these two modules corresponds to the mean membrane potential of *P*_*A1*,*l*_ and *P*_*A1*,*r*_ respectively. Two Gaussian additive noise inputs representing non-specific cortical excitation from local cortical neural populations stochastically modulate the excitability of the principal neuronal populations *P*_*A1*,*l*_ and *P*_*A1*,*r*_ respectively.

#### 2.1.2 The thalamic modules

The architecture of the left (Fig 1C) and right (Fig 1D) thalamic modules follows that of the cortical modules. Each of these modules is formed of a principal cell neuronal population, (*P*_*Th*,*l*_ and *P*_*Th*,*r*_) and two interneuron populations representing fast-mediated (*I*_*Th*,*l*_ and *I*_*Th*,*r*_) and slow-mediated GABAergic transmission (*I’*_*Th*,*l*_ and *I’*_*Th*,*r*_). Likewise, the output of the thalamic modules corresponds to the mean membrane potential of *P*_*Th*,*l*_ and *P*_*Th*,*r*_ respectively.

#### 2.1.3 The brainstem module

The brainstem module is composed of two lumped neuronal subpopulations (Fig 1E): an excitatory principal cell population *P*_*BS*_ and an inhibitory neuronal population *I*_*BS*_. The *P*_*BS*_ population projects excitatory connections to the cortical and thalamic modules and receives in return excitatory feedback from these four modules. A Gaussian additive noise input representing non-specific excitatory local projections stochastically projects to *P*_*BS*_ modulating its excitability. The output of this module corresponds to the mean membrane potential of subpopulation *P*_*BS*_.

#### 2.1.4 The cochlear module

The cochlear module (Fig 1F) is the most important module of this model. It presents a lumped representation of the effects of the cochlear stimulation current on the mean firing rate of the auditory nerve. For simplicity, a single electrode of the CI electrode array is represented in this module. The electrochemical diffusion of the stimulation current is represented by a capacitive electrode-electrolyte (cochlea) interface (see [22] for a review). The transfer function of this interface is expressed as follows:

$$v_{stim}=Z_{ec}∙i_{stim}+R_{s}∙i_{stim}$$ (1)

The potential, *v*_*stim*_, induced by the stimulation current *i*_*stim*_ adds up to the mean membrane potential of the auditory nerve. Its value depends on the impedance of the electrode-cochlea interface, *Z*_*ec*_, detailed in Eq (2) in the form of a transfer function, and the cochlea’s input resistance *R*_*s*_.

$$Z_{ec}=C_{dl}/\left(s+1/Z_{f}C_{dl}\right)$$ (2)

The parameter *C*_*dl*_ designates the double layer capacitance of the electrode cochlea interface while *Z*_*f*_ represents its faradaic resistance.

Then, the resulting average potential, *v*, is transformed into an equivalent mean firing rate via the nonlinear transfer function *S*(*v*).*S*(*v*) is the sigmoidal wave-to-pulse function as described by Freeman [23] and is expressed formally as follows:

$$S(v)=2e_{0}/\left(1+exp(r(v-v_{0}))\right)$$ (3)

where 2*e*_0_ represents the maximal mean firing rate of the auditory nerve, *v*_0_ the corresponding postsynaptic potential at a mean firing rate *e*_0_, and *r* the slope of the sigmoidal function *S*(*v*). Residual hearing was modeled as a Gaussian additive noise input that adds up to the stimulation input of this module. Its parameters are detailed in Table 1 (Appendix–S14 File).

**Table 1:** Mutual information (MI) values between the artifactual source and each of the neural sources simulated by the model. The second column presents the raw estimated values of MI and the third presents the corrected values w.r.t the estimation bias.

Source	Uncorrected MI	Corrected MI
**Brainstem**	0.0086	1.9x10^-4^
**Right Thalamic**	0.0089	5.1x10^-4^
**Left Thalamic**	0.009	5.7x10^-4^
**Right Cortical**	0.0065	≈ 0
**Left Cortical**	0.0070	≈ 0

This module is an essential element for simulating the artifact-response mixture for two reasons. Firstly, the dynamics of the mean membrane potential, induced by *i*_*stim*_, contribute to the simulation of the artifactual component of the EEG dataset. Secondly, the output of this module, representing the mean firing rate of the auditory nerve, is the modulatory input of the simulated temporal dynamics of the five remaining modules. Therefore, this module is the basis for generating the AEP components of the simulated EEG dataset, namely the ASSRs in this study. Consequently, these dynamics depend on the choice of the stimulation current at the input of the cochlear module, which in turn should correspond to the electrical translation of the hypothetical acoustic input.

### 2.2 Model input

CIs map the perceived sound to electrical impulses via a coding strategy. This coding strategy is dependent on the CI manufacturer [24]. Generally, sound intensity is coded by amplitude modulation (AM) or pulse width modulation (PWM). This information is thus transmitted by varying either the amplitude or the duration of the stimulation pulses respectively. On the other hand, the spectral envelope is coded by the spatial activation of intracochlear electrodes, with the most basal ones coding higher frequencies. Finally, the stimulation frequency of the CI is manually tuned patient-wise to optimize perception. It is of fixed value and is usually set in the range of 500 Hz. Recent studies reported that speech perception is enhanced with CI stimulation rates in the range of 500 to 600 Hz compared to lower or higher frequency ranges [25, 26].

In this study, the model input was determined with respect to the particular application explored, i.e. Auditory Steady State Responses (ASSRs) in CI patients equipped with a Digisonic SP cochlear implant (Oticon Medical, Vallauris, France). ASSRs are stationary AEPs elicited by a continuous auditory stimulus [16] generally composed of frequency or amplitude-modulated sine tones. Therefore, the model input can be defined as the corresponding CI stimulation current encoding an AM sine tone stimulus by PWM, in view of the Digisonic SP’s coding strategy. Technically, the input used was a PWM monophasic pulse train with the following parameters: the carrier frequency (*f*_*c*_), also representative of the aforementioned CI stimulation rate, was set to 500 Hz as in the general clinical context, the modulation frequency (*f*_*m*_) was set to 39.06 Hz, and the modulation depth to 75%. The choice of the modulation frequency and the modulation depth aimed at amplifying the response of the auditory nerve and the cortical AEP generators to stimulation. Since the same parameters are to be used with the CI patient, this should enhance the detectability of the response [16]. Monophasic rather than biphasic or charge-balanced current was used in this study for simplicity without loss of generality.

### 2.3 Artifact-response mixture simulation: The forward model

**Fig 2:**
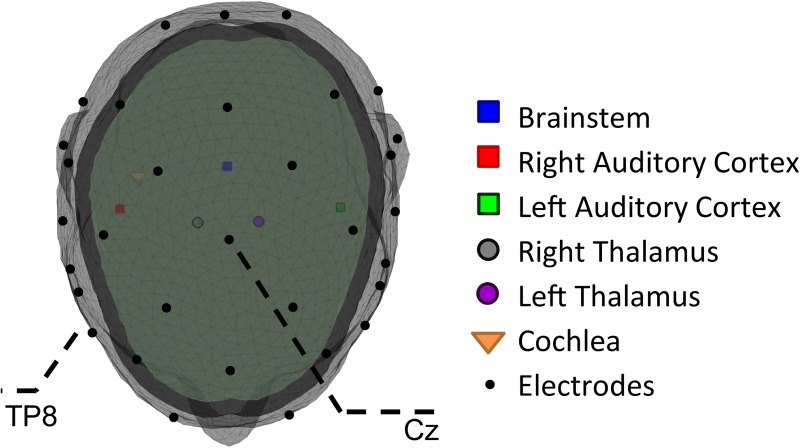
The 3-layered head model and the positions of the 6 dipoles and the 32 electrodes.

The output of each of the six modules of the lumped-parameter model represents the local field potential of each one of them and can be theoretically measured by depth electrodes implanted at these nodes. In order to transform these direct measures into simulated scalp potentials, a realistic 3-layered head model (Fig 2) was constructed in Matlab^®^ using the Fieldtrip toolbox (Donders Institute for Brain, Cognition and Behaviour, Radboud University Nijmegen, the Netherlands. See http://www.ru.nl/neuroimaging/fieldtrip) [27]. This model allows for the estimation of a transfer matrix (or lead field matrix) by using six dipoles each representing one of the ssix modules presented in section (2.1). The lead field matrix models the passive conduction of the electromagnetic field generated by these dipoles. The temporal dynamics of these six dipoles are modeled by the output of their corresponding modules. Dipole orientations and coordinates can be defined application-wise. In this paper, their definition is based on the state-of-the-art ASSR literature [16, 21]. Their orientations were further tuned to match the topographies obtained experimentally in a pilot study. Dipole orientations are then specified in an additional matrix, *K*, of the forward model’s formulation. The respective coordinates of dipole locations were adapted given the template MRI in such a way that the cochlear dipole is positioned in the cochlea, the cortical dipoles in the primary auditory cortices (symmetric), the thalamic dipoles in the thalamus (symmetric) and the brainstem dipole in the brainstem. Dipole parameters are specified in the Appendix (S14 File Table 2). The conductivity ratios of the scalp, the skull and the brain in such 3-shell head models are often set to 1, 1/80, and 1 respectively. The scalp to brain conductivity ratio of 1/80 was first reported by Rush and Driscoll [28] and has been commonly used ever since as a standard value in head models. The conductivities of the scalp and the brain were set to 0.33 S/m [29] and therefore that of the skull to 0.042 S/m.

**Table 2:** Average negentropy of simulated *EEG*_*mix*_ channels and detected sources per algorithm obtained over 10 model simulations (MD = 75%)–see S1 Fig for a boxplot presentation.

	*EEG*_*mix*_ channels	*infomax*	*extended infomax*	*jade*	*fastICA*
Average negentropy	0.1387 ± 1.1 x 10^−4^	7.13 x 10^3^ ± 1.07 x 10^3^	3.75 x 10^3^ ± 1.28 x 10^3^	2.28 x 10^3^ ± 356	4.49 x 10^3^ ± 868

Instead of computing a single lead field matrix accounting for all the modeled dipoles, we computed two separate matrices, the first, *LF*_1_, for the five neural dipoles and the second, *LF*_2_, for the artifactual dipole (cochlea). Once the lead field matrices had been calculated, the direct EEG problem was formulated as follows:

$$EEG_{mix}=LF_{1}∙K_{1}∙S_{n}+LF_{2}∙{K_{2}∙S}_{Art}+b$$ (4)

Matrices *K*_1_ and *K*_2_ define the orientation specifications of the neural and artifactual dipoles respectively. Therefore, the simulated EEG takes into consideration the neural temporal dynamics, represented by matrix *S*_*n*_, as well as the artifactual dynamics related to cochlear stimulation, represented here by matrix *S*_*Art*_. Additive white Gaussian noise is also modeled at the recording electrodes by matrix *b*. Moreover, the contribution of the dipoles of interest to the simulated EEG is computed systematically as follows:

$$EEG_{ASSR}=LF_{1}∙K_{1}∙S_{n}$$ (5)

*EEG*_*ASSR*_ represents the uncontaminated control dataset that is necessary to evaluate the efficiency of artifact suppression algorithms. In this study, the simulated EEG was sampled at 50 kHz (user defined in the model).

Being built on a stochastic neural population model, the output of the forward EEG model presented in this section slightly varies at each simulation without hindering the generation of the basic neural and artifactual dynamics required for this study.

### 2.4 Comparing ICA algorithms

As previously stated in the introduction, our interest in independent component analysis (ICA) is motivated by the temporally widespread stimulation artifact induced by a monopolar unsymmetrical stimulation current in the CI of interest, the Digisonic SP implant. In such a context, the commonest efficient method of CI artifact removal in the context of ASSRs, blanking [11, 18, 19], would require the loss of up to 50% of the recorded EEG signal. Moreover, ICA is another frequently used method to attenuate or remove the cochlear stimulation artifact in CI patients’ EEGs [10, 14, 17, 30] without systematically eliminating segments of the signal of interest. However, ICA involves various algorithms, of various mathematical and computational complexities. In the present study, only the following four popular ICA algorithms were tested for their efficiency in removing the CI artifact: *infomax* [17, 31], *extended infomax* [32], *jade* [33] and *fastICA* [34] given their previous reported usage in denoising the CI artifact [14, 17, 30, 35, 36].

#### 2.4.1 The ICA algorithms in a blink

The four algorithms tested, *infomax*, *extended infomax*, *fastICA and jade*, aim at achieving statistically independent sources following non-Gaussian distributions at their output. However, in order to achieve this statistical independence, these methods employ different criteria, or contrast/likelihood functions.

*Infomax* and *extended infomax* separate a blind mixture by minimizing the mutual information among the extracted sources [31, 32]. Although these two methods stem from the same basic mathematical contrast function, *extended infomax* provides a higher flexibility concerning the statistical distribution of the detected source due to its generalized learning rule. While *infomax* [31] allows the detection of supra-Gaussian sources only, *extended infomax* [32] takes sub- and supra-Gaussian sources into account.

The contrast function of the *fastICA* algorithm is based on maximizing negentropy, which is a measure of the distance between the distribution of the detected source and that of a normal distribution of the same mean and standard deviation [34]. As a matter of fact, it has been shown that maximizing negentropy is mathematically equivalent to minimizing mutual information (see [34]), implying that *infomax*, *extended infomax* and *fastICA* belong to the same family of ICA algorithms. Also, this algorithm takes as input parameter, the type of contrast function used to estimate negentropy [34]. In our simulations, we used the default contrast function, which is a general purpose function based on kurtosis. Finally, *jade* maximizes the kurtosis of the separated sources by jointly diagonalizing their fourth order cumulant matrices [33].

#### 2.4.2 ICA source constraints

In order for ICA algorithms to be applicable, two major constraints should be respected: 1) the statistical independence and 2) the non-Gaussianity of the mixed sources. These two constraints have been validated for EEG signals [37]. Any Gaussian source will be perceived as a mixture of two or more non-Gaussian signals, according to the central limit theorem, and will therefore be impossible to detect as a single independent component. Therefore, proving the non-Gaussianity of our simulated sources is important. Similarly, measuring the statistical independence between the artifactual source and each of the neural sources in our simulations is crucial.

The non-Gaussianity of each source was tested using the Anderson-Darling test (adtest Matlab^®^ statistical toolbox function). This function tests the null hypothesis that the each of the simulated source dynamics follows a normal distribution. Then, the statistical independence between the artifactual source and each of the simulated neural sources was evaluated by calculating the mutual information (MI) based on the Kullback entropy [38] expressed as:

$$MI(X,Y)=\sum_{i,j}p(X_{i},Y_{j})∙log(\frac{p(X_{i},Y_{j})}{p(X_{i})∙p(Y_{j})})$$ (6)

where *X* and *Y* are two random variables. The naïve algorithm, or the histogram based technique was accordingly implemented to estimate *MI*. Also the obtained values were corrected by an estimation bias of $\frac{{\left(M-1\right)}^{2}}{2}∙\frac{1}{N}$ where M is the number of histogram bins and N is the number of samples in each signal [39].

#### 2.4.3 Comparing efficiency

These methods are here compared based on the error of estimation of the amplitudes of the expected simulated responses. A significant increase or decrease in response amplitude observed in the denoised dataset is considered as an indication of undesired performance. In other words, a positive estimation error indicates the presence of artifactual residuals in the denoised dataset. Likewise, a negative estimation error implies an imperfect separation of the artifact-response mixture, leading to the rejection of an artifactual independent component actually containing a neural response.

Artifactual components were identified and rejected manually based on their characteristic topographical and frequency distribution calculated by FFT (*F*_*IC*_). Ten EEG simulations were performed, of 40 s each, and the four ICA algorithms were operated accordingly on each simulation. This aims at taking the stochastic nature of the model and therefore the respective performance of each algorithm into consideration.

More generally, the efficiency of these algorithms can be evaluated as a function of many parameters (stimulation frequency, cochlear dipole position and orientation, etc.) using the model. In this study, the effect of the modulation depth of the input signal on the performance of ICA algorithms was assessed. In fact, a recent study showed the possibility of eliminating the transcranial alternating current stimulation artifact in MEG recordings using beamformers only if an amplitude modulation is applied to the sinusoidal stimulation signal [40]. Therefore, supplementary simulations were performed accordingly for the following values of modulation depth: 100, 75, 50 and 20% in order to evaluate possible effects.

### 2.5 Proof of concept: ICA algorithms in a real dataset

As previously stated, it is quite impossible to assess the efficiency of a CI artifact-denoising algorithm by applying it directly to real datasets recorded in CI patients without taking into consideration the potential effects on the underlying AEPs. However, it is possible to compare the outcome of several denoising algorithms obtained from processing a given CI patient’s EEG dataset. Coupled with the computational results, the clinical exploration can offer insight into the actual effects of these algorithms in real data.

In this section, the patient EEG dataset used to compare and contrast the aforementioned four ICA algorithms is described. This study was approved by the local ethics committee (CPP Sud-Est IV 14/034 ID RCB 2014-A00345-42) and data acquisition was performed with the written informed consent of the patient. The subject is a profoundly deaf male patient, 68 years old, who had been implanted for 2 years at the time of the experiment.

The stimulus was constructed in Matlab^®^ in accordance with the parameters specified in the computational approach. It consisted of an amplitude modulated pure tone with a carrier frequency of 1 kHz, a modulation frequency of 39.06 Hz and a modulation depth of 75%. Note that the choice of the acoustic carrier frequency targeted the activation of the CI electrode coding for the 1 kHz frequency range. Furthermore, the electrical carrier frequency was equivalent to the patient’s CI stimulation rate *f*_*stim*_ set to 500 Hz as in the daily usage protocol. This frequency matches the carrier frequency of the stimulation pulses at the model input. The sound intensity was calibrated to 70 dB SPL. It was then presented acoustically via the auxiliary input of the cochlear implant for a continuous duration of five minutes.

EEG activity was recorded using 31 active electrodes (actiCAP, Brain Products GmbH, Munich, Germany) placed following the 10–10 international system. EEG signals were amplified and digitized at a sampling frequency of 50 kHz (actiCHamp, Brain Products GmbH, Munich, Germany). The recorded data were grounded to the forehead and referenced to the nose. ICA algorithms were applied respectively to this dataset. The number of components was fixed at 31 for all the algorithms. The artifactual component(s) was manually rejected based on its particular scalp topography and its particularly distributed frequency spectrum.

## 3 Results

This section presents the major findings of the study. First, the simulated dynamics are described and their topographical distributions depicted. Then, the ASSR and the artifact dynamics are illustrated as projected at the scalp electrodes. Finally, the denoising results obtained by applying the four tested ICA algorithms to simulated and real data are presented.

### 3.1 From the dynamics of the neural mass model to simulated EEG

**Fig 3:**
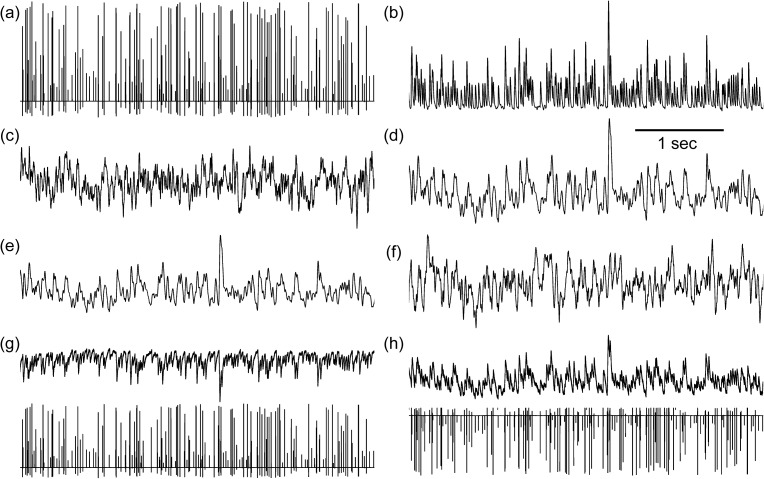
Simulated dynamics (arbitrary units) at the (a) cochlear module, (b) brainstem module, (c) right thalamic module, (d) left thalamic module, (e) right cortical module and (f) left cortical module. Projected scalp potentials at scalp electrodes (g) TP8 and (h) Cz. Upper panels correspond exclusively to the neural contribution and the lower panels represent the contaminated signals.

Due to the capacitive characteristics of the electrode-cochlea interface, the envelope of the model’s PWM input is slightly deformed to follow the modulation frequency *f*_*m*_ (Fig 3A). This signal represents the temporal dynamics of the cochlear artifact dipole. Moreover, the simulations show that the PWM input (*f*_*c*_ = 500 Hz; *f*_*m*_ = 39.06 Hz) generates typical oscillatory dynamics in the neural mass model that can be representative of the simulated ASSRs. As depicted in Fig 3B, the output of the brainstem module conserves the high frequency content of the input signal readily following its envelope (*f*_*m*_). As expected, the induced oscillations are low-pass filtered throughout the thalamic nodes (Fig 3C and 3D) and up to the cortical nodes (Fig 3E and 3F). This is in part due to the explicit modeling of the pulse-to-wave transfer function [23] in neural mass models. However, this may still explain in part the participation of the cortical nodes in ASSRs generated by a modulation frequency around 40 Hz but not around 80 Hz, higher frequencies being filtered throughout the ascending auditory pathways. The dynamics generated at the cortical nodes exhibit considerable low frequency content (< 200 Hz). At this level, the spectral peak at the modulation frequency loses its prominence. This peak can be re-established by averaging the simulated cortical dynamics over fixed-time segments of duration *D* equivalent to an integer number (*k*) of the modulation period (*T*_*m*_), such that *D* = *k* ∙ *T*_*m*_.

On the system level, the simulated EEG datasets, *EEG*_*ASSR*_ and *EEG*_*mix*_, generated using Eqs (4) and (5) and the temporal dynamics simulated by the nodes of the lumped parameter model, show a major contamination of scalp electrodes by the stimulation artifact. Fig 3G and 3H depict the simulated neural-based (*EEG*_*ASSR*_, upper panel) and mixture-based (*EEG*_*mix*_, lower panel) EEG activity recorded at electrodes TP8 and Cz respectively.

### 3.2 Non-Gaussianity and statistical independence

The Anderson-Darling test confirmed the non-Gaussianity of all simulated sources as the null hypothesis, that each considered source follows the normal distribution, was rejected (p < 10^−3^ for all sources). Concerning mutual independence, the number of histogram bins was estimated based on the amplitude intervals of the simulated sources based on Scott’s rule [41]. The smallest (*M* = 160) value obtained over all sources was used in order to minimize estimation bias (section 2.4.2). The uncorrected values of the mutual information between the artifactual source and each of the neural sources are depicted in the second column of Table 1. These values are of the same order of magnitude as the estimation bias (b = 0.0084), therefore indicating extremely small values of mutual information. The corrected values are presented in the third column of Table 1. These values confirm the statistical independence of the artifactual simulated dynamics of those of the neural sources simulated by the model.

Furthermore, MI was also computed between *EEG*_*ASSR*_ and *EEG*_*Art*_ (the simulated EEG dataset accounting for the artifactual source only) for two electrodes, *T8*, representing a highly contaminated electrode, and *Cz*, representing an electrode of high ASSR amplitude. The corrected MI values for both electrodes were smaller than 10^−4^, once again indicating the statistical independence of the artifactual and neural simulated sources.

Finally, the mutual information among the neural sources was computed. It is worth mentioning that almost all the sources presented little mutual information (corrected values < 0.07). The two cortical sources presented a relatively high value of MI (corrected value of 0.1). The thalamic sources (left and right) presented a significantly high value of MI (corrected value of 3.9).

### 3.3 The efficiency of ICA algorithms in silico

**Fig 4:**
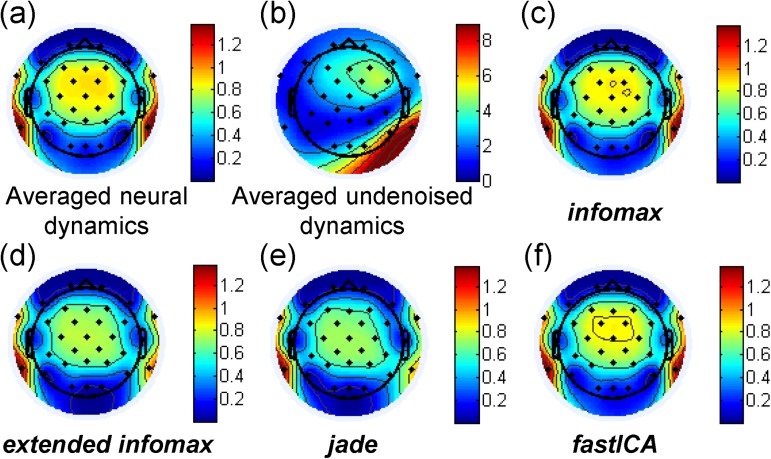
Simulated topographies of ASSR amplitudes (μV) calculated from (a) the neural dataset, (b) the mixed dataset, and the datasets denoised by (c) *infomax*, (d) *extended infomax*, (e) *jade* and (f) *fastICA*.

Fig 4 provides a representative example of the topographies of detected averaged amplitudes obtained from one simulation of the model and the outcome of the four ICA algorithms. Response detection was performed by phase coherence [42]. Phase coherence is a measure of the phase-locking among the occurrences of a defined frequency component in a continuous signal. In this study, each epoch is defined as a time segment containing 20 modulations of the presented acoustic signal. Therefore, whenever an ASSR is evoked due to the presented stimulus, its phase is hypothetically constant over the epochs. According to [42], phase coherence is calculated according to the following equation:

$$PC=\frac{1}{N}∙\sqrt{{\left(\sum_{i=1}^{N}\cos\;\theta_{i}\right)}^{2}+{\left(\sum_{i=1}^{N}\sin\;\theta_{i}\right)}^{2}}$$ (7)

where *θ*_*i*_ is the phase value of the FFT of epoch *i* at the frequency of interest (i.e. the modulation frequency) and N is the number of the considered epochs. When *PC* tends to 1, it indicates perfect phase-locking of the underlying frequency component throughout the epochs. Values inferior to 0.5 indicate poor phase-locking. In this study, a response was only detected when its phase coherence attained a value above 0.8 over several sweeps (in this study predefined as 20 epochs). Fig 4A represents the scalp topography of ASSR amplitudes obtained by averaging the dataset *EEG*_*ASSR*_ over 150 consecutive time segments of duration *D* = *k* ∙ *T*_*m*_, where *k* = 20. The neural dipole orientation parameters were tuned in order to focalize the ASSR distribution over the frontal and central electrodes, notably around Cz in accordance with the current ASSR literature [21] and based on experimental data obtained in normal hearing participants in a pilot study. Similarly, the orientation of the cochlear dipole was also tuned to achieve a realistic topography similar to what is generally observed in implanted patients, i.e. an ipsilateral spread of the stimulation artifact, most intense near the CI implantation site. Note that the topography of contamination and its spread varies slightly from one patient to another in terms of electrodes affected and degree of contamination. The considered simulation parameters present a case of wide-spread contamination affecting the electrodes of the fronto-central region of interest. Fig 4B depicts the topographical distribution of the averaged scalp potential calculated from the mixed dataset *EEG*_*mix*_. These plots show that the artifactual potential is generally maximal around the implantation site but may still contaminate central and frontal electrodes. Also, the order of magnitude of the averaged artifactual potentials is significantly larger than that of the averaged neural potentials (8 μV vs. 1 μV). Equivalent contamination schemes can be observed in real undenoised datasets recorded from CI patients and depending on the orientation of the cochlear dipole. This computationally confirms that averaging raw EEG data without prior denoising would certainly induce artifactual responses. However, the degree and spread of contamination is dependent on the surgical emplacement of the CI and therefore on the position and orientation of the cochlear stimulation dipole. Hence, denoising is an indispensable step ahead of averaging in order to minimize false detection.

The computational results demonstrate that the four ICA algorithms tested, *infomax*, *extended infomax*, *jade* and *fastICA*, efficiently denoise the simulated dataset *EEG*_*mix*_ under the simulation conditions considered in this study (notably high sampling rate). For information, in our simulations, only one component was automatically detected and rejected for *infomax*, *extended infomax*, and *jade* out of 25 computed independent components. The number of computed ICs was limited to 25 in the simulated data to limit the computation time required for convergence. This was sufficient given the simplicity of the modeled dynamics. Further increasing the number of ICs in the model did not enhance the quality of the results. Also, one component was rejected for *fastICA* out of the 4 independent components detected. In fact, the *fastICA* algorithm automatically adapts the number of independent components to be estimated from a given dataset. In the simulated EEG, *fastICA* estimated the number of independent components at 4 components. The topographies of the averaged potentials calculated from the denoised datasets are depicted in Fig 4C–4F. When applying *infomax* (Fig 4C), the amplitudes of the resulting denoised potentials significantly approach those of the averaged neural potentials (Fig 4A), whereas *extended infomax* (Fig 4D) as well as *jade* (Fig 4E) induce a decrease in the response amplitudes over all electrodes, indicating a probable imperfect separation of the artifact-response mixture using these two algorithms. Therefore, part of the response is eliminated with the artifactual component detected. Finally, using *fastICA*, we obtained a topographical representation of the detected responses (Fig 4F) that significantly resembles the topographical distribution of the neural dataset (Fig 4A).

#### 3.3.1 ASSR amplitude estimation error

**Fig 5:**
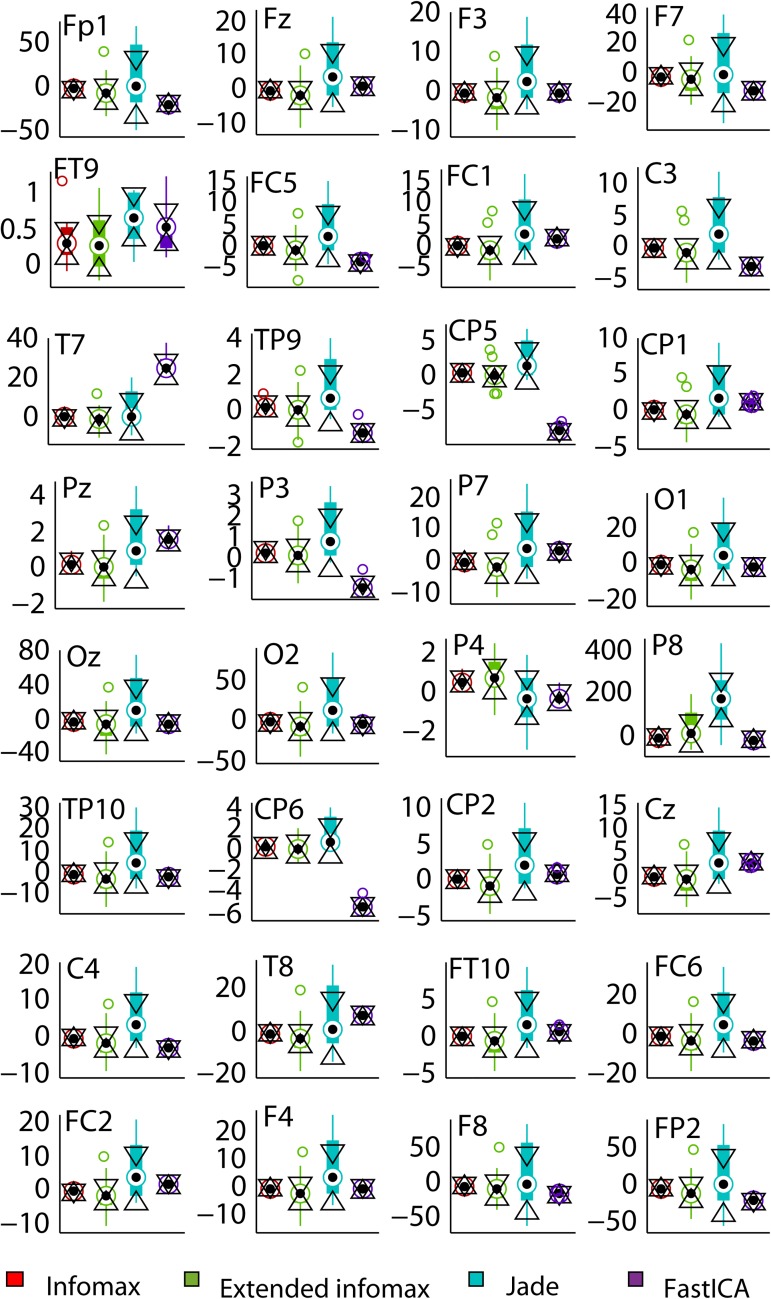
Boxplots of the ASSR amplitude estimation error obtained from 10 denoised datasets after the application of *infomax* (red), *extended infomax* (green), *jade* (blue), *fastICA* (purple) at the 32 scalp electrodes.

Fig 5 depicts the stochastic distribution of the amplitude estimation error (in percentage) induced by each of the tested algorithms as compared to the expected (neural) ASSR amplitudes. These boxplots represent the results of 10 simulations for each method. As observed at all electrodes, *infomax* and *fastICA* present the smallest variance and can therefore be considered to be the most stable among the four algorithms. Yet, the estimation error incurred by *infomax* remains the closest to 0%, thus suggesting that this algorithm retrieves the expected neural ASSR amplitudes at most of the recording electrodes.

To confirm the significance of the error estimation difference among the 4 methods, a region of interest was first defined. This concerned all the fronto-central electrodes, Fz, F3, F7, FC5, FC1, C3, Cz, C4, FC6, FC2, F4 and F8. All 10 repetitions were included in the statistical analysis performed with the anova2 function (Statistical toolbox, Matlab^®^, Mathworks). The choice of the denoising algorithm proved significant in determining the estimation error (ANOVA: F(3,432) = 76.13, MSE = 3557.67, p < 10^−4^). Post-hoc Tukey’s HSD tests showed that the estimation error obtained over all the electrodes considered was significantly minimal (mean value = -2%) when computed by *infomax* as compared to the other four algorithms. Also, the estimation errors of *extended infomax* and *fastICA* were not significantly different for this set of electrodes. Yet, it is worth mentioning, that the estimation error was significantly different between *infomax* and *fastICA* over 80% of the electrodes considered when individually tested using ANOVA (p < 10^−3^). Also, the outcome of extended infomax was significantly more variable than that of infomax (ANOVA on estimation error standard deviation; p < 10^−2^).

As for the computational complexity, *infomax* was the most efficient, converging in an average time of 4.7 (standard deviation 0.2397) seconds. Given that execution time depends on the PC used, the average time needed for *infomax* to converge will be denoted as $\hat{t_{conv}}$. *Extended infomax* required on average $400∙\hat{t_{conv}}$ before converging while *jade* converged in approximately $6.96∙\hat{t_{conv}}$ and fastICA in $2.62∙\hat{t_{conv}}$.

In conclusion, this computational investigation advocates the use of *infomax* given its stability (low variability of the estimation error), its precision in amplitude estimation (error closest to 0%) and its low computational complexity compared to the other algorithms. It is thus the most suitable for this context in comparison to the other 3 ICA algorithms tested and under the simulation parameters considered.

#### 3.3.2 Effects of the modulation depth

**Fig 6:**
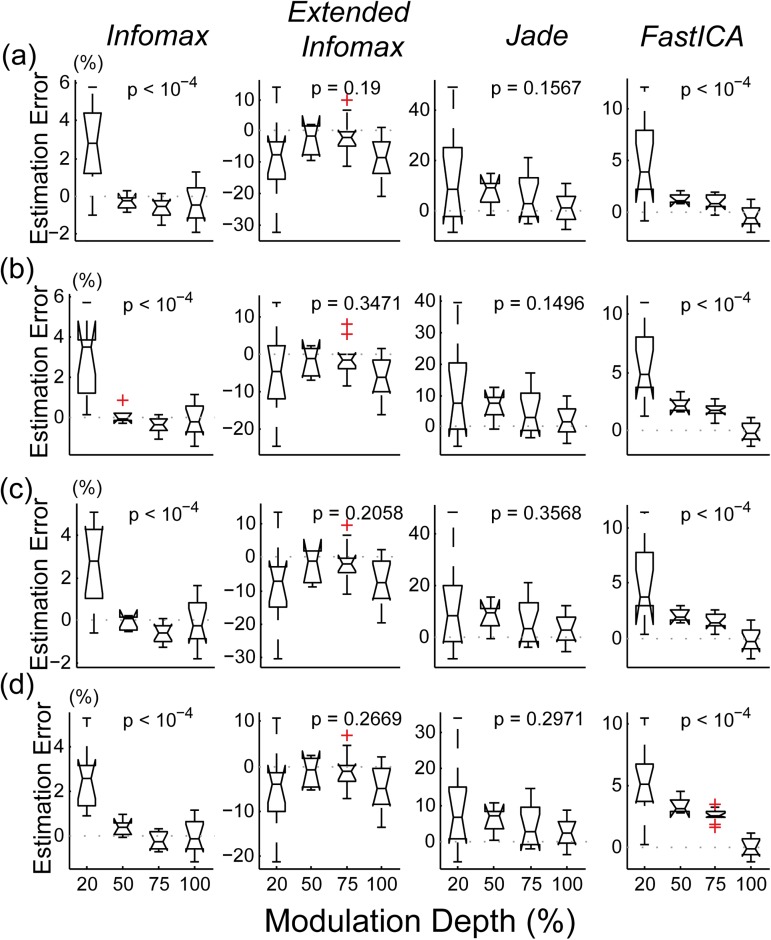
Effect of the modulation depth on the estimation error of the ICA algorithms tested as measured on electrodes Fz (a), FC1 (b), FC2 (c) and Cz (d). The corresponding p values are indicated in the plot.

As expected, the computational simulations showed that varying the modulation depth (MD) of the model input has an effect on the performance of the ICA algorithms (Fig 6). For all four algorithms, the ASSR amplitude estimation error increased as the modulation depth diminished. Fig 6 depicts this change at electrodes Fz (Fig 6A), FC1 (Fig 6B), FC2 (Fig 6C) and Cz (Fig 6D) respectively.

The choice of electrodes can also be justified by the peak values of ASSR amplitudes at these electrodes and their clinical relevance as observed in a pilot study. The represented boxplots indicate that *extended infomax* presents the least variability as a function of MD (Fig 6 second column) and still has a considerable error interval at 100% MD. On the other hand, the performance of *infomax* varies significantly (for example on electrode Cz, ANOVA F(3,31) = 20.32; MSE = 15.49,p < 10^−4^) as a function of the MD (Fig 6 first column). Despite this variability the estimation error remains confined to the interval [-2; 6%]. *Jade* presents the highest sensitivity to MD as evidenced by the high variability of its performance (Fig 6 third column). This variability remains statistically insignificant however, thus reflecting an undesired performance for all MD values. *Jade* can induce up to 40% estimation error when used with low MDs. *FastICA* follows the same behavior as *infomax* (Fig 6 fourth column). However, the interval of estimation error remains slightly wider as it extends over [-2; 10%]. Furthermore, the mean of the estimation error only approaches 0% at 100% MD.

#### 3.3.3 Effects of ICA application on the non-Gaussianity of the estimated sources

Since the maximization of non-Gaussianity is a clear indicator of the performance of an ICA algorithm, the effect of the chosen algorithm on the non-Gaussianity of the separated sources was estimated. Negentropy was chosen as a measure of non-Gaussianity and was calculated based on the estimate provided in [43] and expressed as follows:

$$J(y)={\left(E\{G(y)\}-E\{G(g)\}\right)}^{2}$$ (8)

where *y* is the considered signal, and *g* is a Gaussian variable of zero mean and unit variance, and *G* is a non-quadratic function expressed as follows:

$$G(u)=\frac{1}{a}\log\;\cosh\left(a∙u\right)$$ (9)

and 1 ≤ *a* ≤ 2. In our simulations, *a* was fixed to 1. Negentropy reflects the distance between a given distribution and the normal distribution [43]. The further the value of negentropy from zero, the higher the non-Gaussianity of the concerned distibution.

For 10 model simulations (MD = 75%), the values of negentropy were calculated and averaged over the 32 channels of *EEG*_*mix*_, over the 25 sources detected by each of *infomax*, *extended infomax* and *jade*, and over the 4 sources detected by *fastICA*. Table 2 depicts the average of the obtained values per method as well as the standard deviation. These results are also presented in the form of boxplots in S1 Fig. In fact, the application of each of the ICA algorithms incurred a significant (ANOVA; p < 10^−4^) increase of the order of 10^4^ times in average negentropy among the *EEG*_*mix*_ channels simulated and the sources estimated. This increase in negentropy translates into an increase in non-Gaussianity and therefore validates the convergence of the ICA algorithms applied to the simulated data. Moreover, the increase in negentropy proved to be significantly (ANOVA; p < 10^−4^) the highest for solutions obtained by *infomax* in comparison to each of the other algorithms. This can further corroborate its higher efficiency in comparison to the other algorithms tested in the context of our study.

### 3.4 ICA outcome in CI patient EEG recording

#### 3.4.1 The extracted artifact

**Fig 7:**
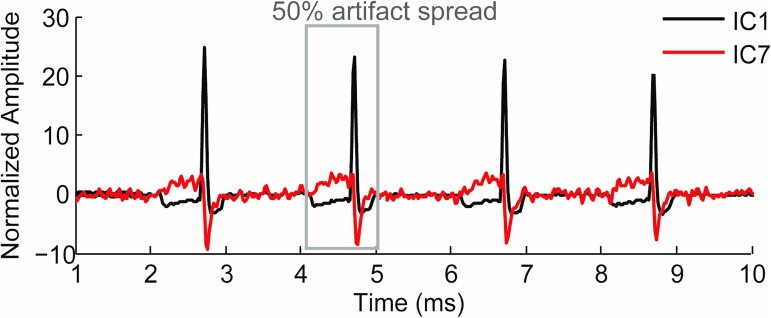
The temporal artifactual spread as observed in real data. CI1 and CI7 are two of the independent components rejected by *infomax*.

As an illustration, Fig 7 shows two of the artifactual independent components rejected by *infomax*. These two major components reflect the artifact’s temporal spread, which is about 50% for this patient. Note that this percentage may vary from one patient to another due to the variability of the subjective pulse duration among patients. This wide temporal spread of the artifactual contamination justifies once again the need to find an alternative method to blanking for this type of stimulation.

#### 3.4.2 The resulting topographies

**Fig 8:**
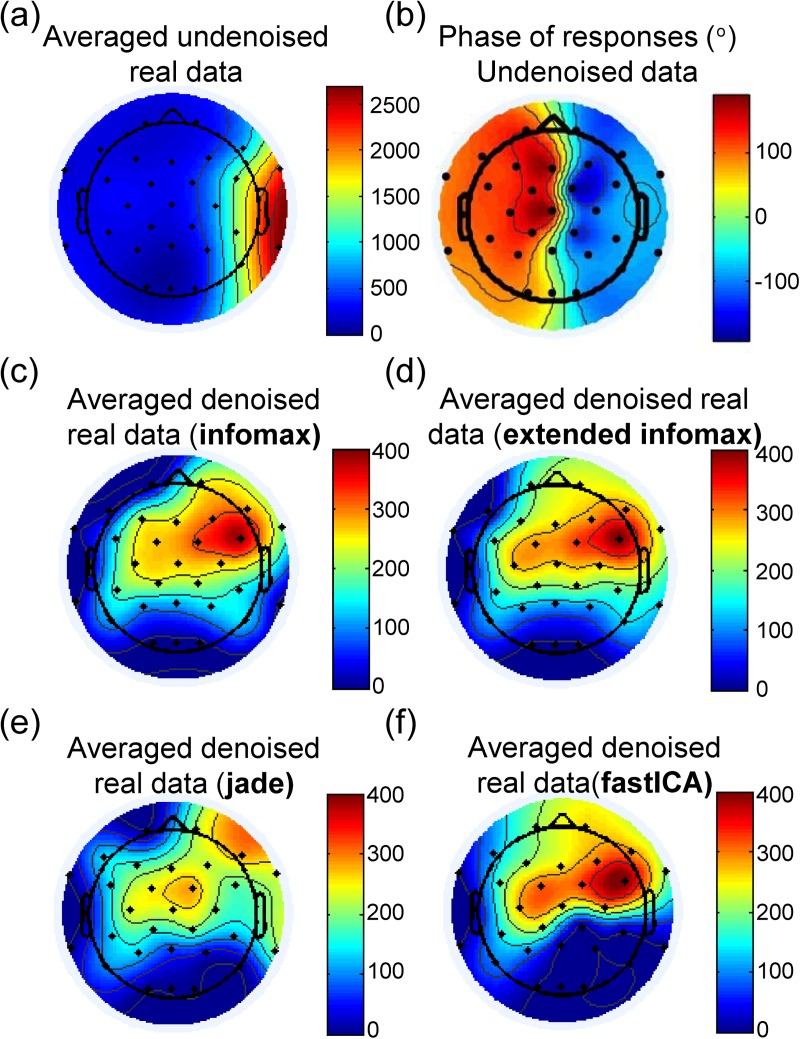
ASSR amplitudes in nV obtained from the undenoised (a) real dataset and their corresponding (b) phase values. ASSR amplitude topographies (nV) of a real CI patient recording denoised by (c) infomax, (d) extended infomax, (e) jade and (f) fastICA.

Applying the different ICA algorithms to the EEG recording of a CI patient (section 2.5) resulted in slightly different estimations of the ASSR amplitude topography among algorithms (Fig 8). The presented topographies color-code only the detected response amplitudes. In the absence of response detection at certain electrodes (e.g., Oz), the response amplitude is color-coded with a zero amplitude response (dark blue). This implies that the phase coherence of the detected amplitude is not significant of sufficient phase-locking. For precision, a maximum of 20% of the estimated components (i.e., 5 components out of 31) was allowed in manual rejection for every algorithm. Namely, 5 components were rejected for *infomax* and *extended infomax*, 4 for *jade*, and 3 for *fastICA*.

Fig 8A represents the topography of the amplitudes of the detected responses obtained from a patient’s undenoised dataset. Note that the visual inspection of this patient’s data showed negligible contamination at electrodes Fz, Cz, Pz and Oz. The phase values of the detected responses are depicted in degrees in Fig 8B. The phase value for this type of stimulation cannot be easily used to confirm or reject the quality of the denoised results as is the case with amplitude modulated biphasic symmetric stimulations [18]. This can be explained by the morphology of the artifact (Fig 7), i.e. the passive components will contribute more or less to the contamination at a given electrode as a function of its distance from the implantation site. Contralateral electrodes exhibit only sharp peaks of contamination as the amplitude of the other two passive phases of the artifact will be of the same order of magnitude as that of the neural activity. The contamination schemes can also vary by the variation in the implantation coordinates and the orientation of the stimulation dipole.

In this study, we did not aim at reproducing computationally the topographies of the recorded patient, but at investigating the subtle variation in ASSR amplitude estimation as a function of the denoising method used. By analogy with the computational results, the equipotential lines of the topographies of *infomax* (Fig 8C), *jade* (Fig 8E), and *fastICA* (Fig 8F) did not present significant differences from those obtained in the model. In other words, the measured ASSR amplitudes were of the same order of magnitude for *infomax* (Fig 6A) and *fastICA* (Fig 6D). The topography obtained with *jade* (Fig 8E) showed a slight decrease in ASSR amplitudes in comparison with the model’s prediction. Finally, the response amplitude topography measured after denoising by *extended infomax* (Fig 8D) did not present a deviation from the *infomax* topography as predicted by our computational framework. This discrepancy can be explained by the high variability in the performance of *extended infomax* for the given recording conditions (MD = 75%). In conclusion, it can be assumed that the order of the induced amplitude change predicted by the model remains credible for the four ICA algorithms tested.

## 4 Discussion

Despite the fact that EEG signal preprocessing (filtering, denoising, averaging, etc.) is an inevitable step for the enhancement of the signal-to-noise ratio in the context of AEPs, the misuse of preprocessing can lead to the deformation and therefore to the misinterpretation of actually evoked responses [44–46].

### 4.1 The computational framework

The major contribution of this paper is the computational tool that it introduces as a means of assessing the effects of preprocessing on raw neural data. This is especially important in the context of cochlear implants given the additional artifactual noise induced by the CI stimulation. Despite its relatively simple architecture, the proposed computational framework allows for the simulation of the artifact-response mixture in a controlled and predictable manner. Thus it permits the evaluation of the effectiveness of the preprocessing algorithm used by comparing the resulting denoised AEPs to the original uncontaminated AEPs. In certain applications, this approach can be impossible to achieve in the clinical setup given the lack of *a priori* information concerning the spread and the characteristics of the CI artifact in a given CI patient.

#### 4.1.1 Simulating the artifactual contamination

The model takes into consideration the six main contributors to the artifact-response (ASSR) mixture in CI patients. This model deliberately models artifactual contamination by stimulation pulses and ignores the representation of the radio-frequency transmission artifact induced by the processor’s antenna for the following reasons. First, the frequency of the RF signal is 4 MHz which is not likely to contaminate responses at the frequency of interest of 39 Hz. Second, even when this signal occurs every time a stimulation pulse is active, it would affect the frequency range around the stimulation frequency and not the modulation frequency. Third, we did not observe unidentified artifactual components in our dataset that could be representative of the RF-induced DC offset artifact [11, 19]. The two DC phases surrounding the active stimulation pulse in the artifactual component IC1 (Fig 7) correspond to two passive stimulation phases of the CI aiming at balancing the Faradaic charge injected by the active pulse.

#### 4.1.2 Construction of the head model

Concerning the head model, the values of layer conductivities used date back to the first reported study on the matter which determined the brain scalp conductivity ratio to be of the order of 1/80 [28]. Although more recent studies report varying values of this ratio mostly dependent on the method of measurement (see [47] for a review), we assume that these values will not drastically affect the simulations of this computational framework as a tool for comparing denoising algorithms. This ratio remains user defined in our implementation.

#### 4.1.3 Relevance of modeling to testing denoising algorithms

To our knowledge, neural mass models and forward EEG models have never been proposed or used before for studying CI artifact denoising algorithms. However, it is worth noting that simulated data have already been exploited to evaluate the performance of denoising algorithms in eliminating artifacts of physiological (muscular, ocular, etc.) and non-physiological origin (mostly related to technical issues) from EEG recordings (see [48] for a review). For instance, Makeig and his colleagues [49] simulated realistic 6-channel ERP data by injecting a noise-free 4-channel ECoG recording into 4 simulated dipolar sources of a 3-layer spherical head model in order to evaluate the performance of ICA in separating the simulated mixture. Also, in order to assess the performance of ICA algorithms in eliminating muscular artifacts from EEG recordings, a realistic 3D head model (3 layers) was used to generate realistic EEG datasets from dipolar sources by solving the forward problem [50].

### 4.2 The tested algorithms

Concerning the particular application of ASSRs, our results show that CI artifact denoising is an important step before detecting responses. Moreover, the particular use of ICA can be an alternative to blanking [11, 18, 19] in patients wearing the Digisonic SP CI (Oticon Medical, Vallauris, France). Otherwise, artifactual responses of relatively high amplitudes (of the order of microvolts) may be detected. In this case, other methods should be deployed for the identification of false alarms. Previous studies used the non-linearity of the amplitude growth function [12] and/or the phase value of artifactual responses [11] to identify non-artifactual responses. For these methods, determining whether a detected response is a false alarm is bound to a comparison with other detected responses at different intensities [12] or at different modulation frequencies [11]. These types of classification may then require several points of measurement to confirm detection.

#### 4.2.1 The algorithms’ performance *in silico*

Nevertheless, the choice of the denoising algorithm has also an effect on the responses obtained. In the results section, it is computationally demonstrated that the performance of four common ICA algorithms (*infomax*, *extended infomax*, *jade* and *fastICA*) in attenuating the CI stimulation artifact is different. Computationally, *infomax* is the most efficient method in reconstituting the expected response amplitudes and remains stable for varying levels of modulation depths. *FastICA* presents a similar stability but tends to incur a negligible increase (< 4%) in the expected amplitude at 75% MD. On the contrary, the *extended infomax* and *jade* algorithms present a significant variability in their outcome. On average, the estimation error of *extended infomax* is centered on 0% with a probable deviation of about ±10% at 75% MD as depicted in the second column of Fig 6. Similarly, *jade* has also a wide variability in its outcome ([-5; 20%]) at 75% MD (Fig 5 and Fig 6) but will on average incur an overestimation of the response amplitude.

#### 4.2.2 The difference in algorithm efficiency

The variability in performance observed among the four algorithms tested computationally in this paper is most probably due to the slight difference in the mathematical approach that they use to optimize statistical independence. For instance, as *infomax* and *extended infomax* use mutual information to estimate independence, their mean output is not statistically different. However, given that the source of interest, the artifact, follows a supra-Gaussian distribution (kurtosis 74.4), the infomax algorithm proves highly sufficient to isolate it. While *extended infomax* takes into consideration sub- and supra-Gaussian sources increasing its computational complexity (i.e. convergence time), its outcome presents significantly more variability than that of infomax (ANOVA test on mean error standard deviation per electrode; p = 0.002). Since extracting sub-Gaussian sources was not part of the studied application, the *extended infomax* algorithm did not provide an enhancement to the *infomax* algorithm.

On the other hand, the performance of the *fastICA* algorithm *in silico* seems to be also justified by the particular contrast function used to estimate statistical independence, negentropy, which is also equivelant to mutual information (see section 2.4.1). This algorithm initializes by estimating the number of independent components to retrieve, 4 in our simulations. This is probably due to the interdependencies between the two thalamic sources as well as the two cortical sources (see section 3.2), which reduces the number of independent elements to 4. Furthermore, the slight positive error in ASSR amplitude estimation (< 4%) may be attributed to the choice of the mathematical contrast function used to estimate negentropy (kurtosis based). As a matter of fact, this general purpose function is kurtosis-based and best suits sub-Gaussian sources [34]. It is not optimal for separating supra-Gaussian sources [34], which is the case of the simulated artifactual source. According to the authors of [34], a Gaussian-based contrast function is better suited to highly supra-Gaussian sources.

Finally, the distinct performance of the *jade* algorithm can be attributed to its distinct core algorithm, which seeks to achieve the statistical independence of its output sources by maximizing their kurtosis through the joint diagonalization of their fourth order cumulant matrices. The resulting separated sources in our simulations do not seem to achieve a clear separation of the artifactual and the neural sources based on this approach.

#### 4.2.3 Performance on real data

In this study, we did not aim at reproducing the exact values of the patient’s ASSR amplitudes in the model. We basically investigated the performance of four commonly used ICA algorithms in separating the artifact from the neural data *in silico* and then we investigated the outcome of these algorithms in a CI patient’s recordings. Furthermore, the results established on the EEG recording of a CI patient proved in line with the computational results. The algorithms *infomax* and *fastICA* yielded similar topographies in the computational simulations (Fig 4C and 4F). The similarity of outcome can be also observed in the real data (Fig 8C and 8F). The *jade* algorithm (Fig 8E) slightly diminished the amplitude topography in comparison with what was observed for *infomax* (Fig 8C). As for *extended infomax*, the algorithm worked quite well on this recording, yielding its average outcome and inducing a comparable topography to that of *infomax* (Fig 8C). More importantly, the variation in the obtained outcome may not be crucial at a stimulation of 75 dB SPL. However, it can play an important role at threshold detection. Therefore, optimal artifact attenuation is of considerable importance.

#### 4.2.4 The use of ICA for CI artifact denoising

ICA algorithms are rarely compared for CI artifact suppression in the context of AEPs using real datasets. And when compared [30], it is generally to test the detectability of an uncontaminated AEP independent component. Conversely, our approach aims at detecting and suppressing artifactual components in CI patient raw data ahead of applying preprocessing (averaging, filtering, etc.) for AEP detection. Although Castaneda-Villa [30] reported that it was not possible to retrieve an uncontaminated AEP independent component using *extended infomax*, it remains the most popular ICA algorithm for CI artifact attenuation [10, 14, 51] followed by *infomax* [36, 52] and then *jade* [35]. Regardless, it is worth noting that the performance of a denoising algorithm is also related to the targeted application and the recording conditions (sampling frequency, number of electrodes, etc.). While many studies [10, 14, 17, 35, 36, 51, 52] successfully used ICA algorithms to extract auditory brain responses from CI patient data, some others failed [53]. The possible absence of statistical independence between the CI artifact and the neural responses due to their time-locking was indicated as a possible reason behind the malfunctioning of ICA algorithms in some applications [54]. Although this might relatively be true, the statistical independence of the stimulation artifact and the relevant neural response (the ASSR in our study), seems to go beyond the notion of time-locking or phase-locking. As a matter of fact, the choice of the recording parameters (sampling frequency, anti-alaising filters …) plays an important role. Applying these preprocessing procedures may deform the electrical stimulation artifact (sampling rate of 125 kHz), extracting its envelope, thus necessitating an extra interpolation procedure to attenuate the artifactual residue after low-pass filtering as illustrated by [13]. In some cases, the artifact residue is more correlated with the neural response than the artifact itself. This is particularly true for ASSRs, sinusoid-like responses time-locked to the stimulus whose envelope is also a sinusoid of the same frequency as the expected response. Therefore, the use of low sampling frequencies at acquisition may hinder the correct application of ICA. Furthermore, it was reported that the ICA algorithm *infomax* successfully denoised the CI artifact in CI-patient ASSR recordings; the sampling frequency used was 8192 Hz [17]. Moreover, using our computational framework, we show that the modulation depth of the stimulus also affects the performance of the ICA algorithms considered in the context of ASSRs. Finally, it is worth noting that spatial filters, such as beamformers, are inapplicable in the case of ASSRs as they require response-free artifact-only time windows to function properly, i.e., a time window in which the neural response is not yet generated. These algorithms are therefore mostly efficient for CI artifact suppression in the context of late AEPs [55].

In conclusion, given 1) the fast convergence of *infomax*, 2) its conservation of the original neural topography (Figs 4A) and 3) the stability of its outcome (Figs 5 and 6), it is advised to privilege the use of *infomax* in the context of ASSRs if an ICA algorithm is to be used. The algorithm *fastICA* may be also considered although its convergence remains bound to the choice of the initial conditions.

## 5 Conclusion

In CI research, the development of objective auditory measures in CIs constitutes a topic of major interest given its impact on the overall functioning of the CI. Not only is the topic important for optimizing the CI parameters but also for preparing progress towards intelligent user-controlled CIs. In this view, it is recognized that the CI stimulation artifact impedes the recording of reliable short and middle-latency AEPs due to stimulation-induced contamination at the recording electrodes. Therefore, denoising and pre-processing constitute two inevitable steps preceding the detection of objective measures (responses) in CI patients. We present an original tool for assessing the theoretical performance of denoising algorithms and their impact on the evoked responses as a function of the simulation parameters. This tool is a computational framework allowing for the simulation of the CI artifact-response mixture of CI patients in a controllable environment. In this work, its utility is illustrated through the study of the efficiency and stability of four ICA algorithms in denoising the CI artifact. Other examples may include the study of the impact of the orientation and the position of the cochlear stimulation dipole in the head model on the performance of denoising algorithms.

Other denoising algorithms that may also be interesting and adapted to PWM asymmetric stimulation are parametric fittings and non-parametric fittings, such as exponential fitting and linear interpolation [9, 13]. In order to study such methods, the integrity of the stimulation artifact should be represented in the model, i.e. including the passive discharge.

At this stage, the current architecture of the model does not allow for the study of the performance of AEP detection algorithms. A possible future solution would be the incorporation of distributed sources of cortical background activity [56]. This would ensure the representation of the cerebral background noise irrelevant to auditory processing. While this enhancement is not necessary for the current purpose of the model, i.e. the study of denoising algorithms, it becomes essential if the latter is to be used to evaluate the efficiency of AEP detection algorithms.

## Supporting information

S1 File Sample simulated EEG data—neural sources (EEGlab Format). (MAT)

S2 File Sample simulated EEG data–artifactual source (EEGlab Format). (MAT)

S3 File Sample simulated EEG data–all sources included (EEGlab Format). (MAT)

S4 File Simulated amplitude estimation error for the algorithms tested at 100% MD. (XLSX)

S5 File Simulated amplitude estimation error for the algorithms tested at 75% MD. (XLSX)

S6 File Real EEG segments used to calculate the mixing matrix for the four algorithms. (MAT)

S7 File Phase values obtained for undenoised patient data (Fig 8B). (XLSX)

S8 File IC1 and IC7 rejected by infomax (Fig 7; sampling frequency 50 kHz). (MAT)

S9 File FFT epoch-wise obtained after denoising by infomax (real data). (MAT)

S10 File FFT epoch-wise obtained after denoising by extended infomax (real data). (MAT)

S11 File FFT epoch-wise obtained after denoising by jade (real data). (MAT)

S12 File FFT epoch-wise obtained after denoising by fastICA (real data). (MAT)

S13 File Frequency axis definition to be used to visualize the FFTs (S9 File–S12 File). (MAT)

S14 File Paper Appendix. Model parameters and details.
(PDF)

S1 Fig Effects of ICA algorithm on the non-Gaussianity of the estimated sources. The depicted boxplots illustrate the average negentropy obtained for the channels of *EEG*_*mix*_ as well as for the estimated sources by each of the ICA algorithms tested (n = 10 simulations). The mean values and their standard deviations are given in Table 2 of the manuscript.
(TIF)
