# Supplementary material for: Auditory steady state responses and cochlear implants: Modeling the artifact-response mixture in the perspective of denoising
Source: PLoS One. 2017 Mar 28;12(3):e0174462. doi: 10.1371/journal.pone.0174462 (PMC5370129; doi:10.1371/journal.pone.0174462)
Supplement: S14 File — Model parameters and details. (PDF) [file pone.0174462.s014.pdf]

Appendix

This appendix provides a list of model's parameters, their (bio-) physical interpretation and their values. These parameters were manually tuned to guarantee the generation of ASSR-like oscillations at the output of the model's modules.

Table 1. Lumped-parameter model parameters

Parameter	Value	Interpretation
A_C	5 (mV)	Amplitude of the cortical average EPSP (cortical modules)
B_C	15 (mV)	Amplitude of the cortical average IPSP ($GABA_{A,slow}$ mediated currents)
G_C	15 (mV)	Amplitude of the cortical average IPSP ($GABA_{A,fast}$ mediated currents)
A_{Th}	5 mV	Amplitude of the thalamic average EPSP
B_{Th}	15 mV	Amplitude of the thalamic average IPSP ($GABA_{A,slow}$ and $GABA_B$ receptors)
G_{Th}	15 mV	Amplitude of the thalamic average IPSP ($GABA_{A,fast}$ receptors)
A_{BS}	5 mV	Amplitude of the brainstem average EPSP
B_{BS}	15 mV	Amplitude of the brainstem average IPSP
$1/a_{CR}$	1/180 s	Time constant of right cortical glutamate-mediated synaptic transmission
$1/b_{CR}$	1/60 s	Time constant of right cortical GABA-mediated synaptic transmission ($GABA_{A,slow}$ receptors)
$1/g_{CR}$	1/160 s	Time constant of right cortical GABA-mediated synaptic transmission ($GABA_{A,fast}$ receptors)
$1/a_{CL}$	1/120 s	Time constant of left cortical glutamate-mediated synaptic transmission
$1/b_{CL}$	1/40 s	Time constant of left cortical GABA-mediated synaptic transmission ($GABA_{A,slow}$ receptors)
$1/g_{CL}$	1/100 s	Time constant of left cortical GABA-mediated synaptic transmission ($GABA_{A,fast}$ receptors)
$1/a_{Th}$	1/120 s	Time constant of thalamic glutamate-mediated synaptic transmission
$1/b_{Th}$	1/40 s	Time constant of thalamic GABA-mediated synaptic transmission ($GABA_{A,slow}$ and $GABA_B$ receptors)
$1/g_{Th}$	1/100 s	Time constant of thalamic GABA-mediated synaptic transmission ($GABA_{A,fast}$ receptors)
$1/a_{BS}$	1/250 s	Time constant of brainstem glutamate-mediated synaptic transmission
$1/b_{BS}$	1/35 s	Time constant of brainstem GABA-mediated synaptic transmission ($GABA_{A,slow}$ and $GABA_B$ receptors)
v_0, e_0, r	$v_0 = 6$ mV, $e_0 = 2.5$ s^{-1} $r = 0.56$ mV^{-1}	Parameters of the nonlinear sigmoid function (transforming the average membrane potential to an average density of action potentials)
C	135	Connectivity constant for the brainstem, the thalamic, the

		right and the left cortical modules respectively
C_1	*C*<br>*(set to 0 in the thalamic module)*	Local collateral excitation connectivity constant
C_2	*0.8·C*<br>*(set to 0 in the thalamic module)*	Local collateral excitation feedback constant
C_3	*0.25·C*	Pyramidal to primary interneuron subpopulation connectiviy constant
C_4	*0.25·C*	Primary interneuron to pyramidal subpopulation connectiviy constant
C_5	*0.3·C*	Pyramidal to secondary interneuron subpopulation connectiviy constant (invalid for the BS module)
C_6	*0.1·C*<br>*(set to 0 in the thalamic module)*	Secondary to primary interneuron subpopulation connectivity constant (invalid for the BS module)
C_7	*0.9·C*	Secondary interneuron to pyramidal subpopulation connectiviy constant (invalid for the BS module)
C_{BS-CR}	10	Connectivity constant from the brainstem to the right cortical module
C_{BS-CL}	5	Connectivity constant from the brainstem to the left cortical module
C_{CR-BS}	10	Connectivity constant from the right cortical to the brainstem module
C_{CR-CL}	5	Connectivity constant from the right to the left cortical module
C_{BS-ThR}	120	Connectivity constant from the brainstem to the right thalamic module
C_{BS-ThL}	120	Connectivity constant from the brainstem to the left thalamic module
C_{CR-ThR}	50	Connectivity constant from the right cortical to the right thalamic module
C_{CR-ThL}	30	Connectivity constant from the right cortical to the left thalamic module
C_{CL-ThR}	0	Connectivity constant from the left cortical to the right thalamic module
C_{CL-ThL}	50	Connectivity constant from the left cortical to the left thalamic module
C_{ThR-BS}	45	Connectivity constant from the right thalamic to the brainstem module
C_{ThL-BS}	30	Connectivity constant from the left thalamic to the brainstem module
C_{ThL-CL}	105	Connectivity constant from the left thalamic to the left cortical module
C_{ThR-CR}	80	Connectivity constant from the right thalamic to the right cortical module
G_{BS}	0.5	Amplification factor of the nonspecific white noise input at the brainstem module
μ_{BS}	0.1	Mean of the nonspecific white noise input at the brainstem module

σ_{BS}	0.1	Standard deviation of the nonspecific white noise input at the brainstem module
G_C	0.5	Amplification factor of nonspecific cortical noise input
μ_C	0.1	Mean of the nonspecific cortical noise input
σ_C	0.1	Standard deviation of nonspecific cortical noise input
G_{RH}	$0.75 \cdot G_{BS}$	Amplification factor of nonspecific cochlear noise input
μ	0.1	Mean of nonspecific cochlear noise input
σ	0.1	Standard deviation of nonspecific cochlear noise input
Z_f	2 kΩ	Faradaic impedence at the electrode-cochlea interface
C_{dl}	24 nF	Double layered capacitance at the electrode-cochlea interface
R_S	20 Ω	Input resistance at the electrode-cochlea interface
f_{stim}	500.5 Hz	Stimulation rate of cochlear current (equivelant to the carrier frequency in this study).
f_m	39 Hz	Modulation frequency of the input current
MD	100, 75, 50, 25%	Modulation depth of stimulation input current
MaxP	40μs	Maximum pulse width of input current

Table 2. Head model dipole parameters (CTF coordinates)

Dipole Parameter	Value	Interpretation
(Y_{CI}, X_{CI}, Z_{CI})	(3.5, -42.5, 12.5)	Coordinates of the cochlear dipole
(Y_{BS}, X_{BS}, Z_{BS})	(-1, 0, 4)	Coordinates of the brainstem dipole
$(Y_{ThR}, X_{ThR}, Z_{ThR})$	(25.5, -14.5, 48.5)	Coordinates of the right thalamic dipole
$(Y_{ThL}, X_{ThL}, Z_{ThL})$	(25.5, 14.5, 48.5)	Coordinates of the left thalamic dipole
(Y_{CR}, X_{CR}, Z_{CR})	(18.5,-51.5,60.5)	Coordinates of the right cortical dipole
(Y_{CL}, X_{CL}, Z_{CL})	(19.5,53.5,54.5)	Coordinates of the left cortical dipole
$(\theta_{CI}, \varphi_{CI})$	(2π/18, 88π/180)	Orientation angles of cochlear dipole
$(\theta_{BS}, \varphi_{BS})$	(π/10, π/2)	Orientation angles of brainstem dipole
$(\theta_{CR}, \varphi_{CR})$	(π/100, -π/3)	Orientation angles of right cortical dipole
$(\theta_{CL}, \varphi_{CL})$	(π/100, 4π/3)	Orientation angles of left cortical dipole
$(\theta_{ThR}, \varphi_{ThR})$	(π/100, π/4)	Orientation angles of right thalamic dipole
$(\theta_{ThL}, \varphi_{ThL})$	(π/100, 3π/4)	Orientation angles of left thalamic dipole
