# Supplementary figures and images for: Auditory steady state responses and cochlear implants: Modeling the artifact-response mixture in the perspective of denoising
Source: PLoS One. 2017 Mar 28;12(3):e0174462. doi: 10.1371/journal.pone.0174462 (PMC5370129; doi:10.1371/journal.pone.0174462)

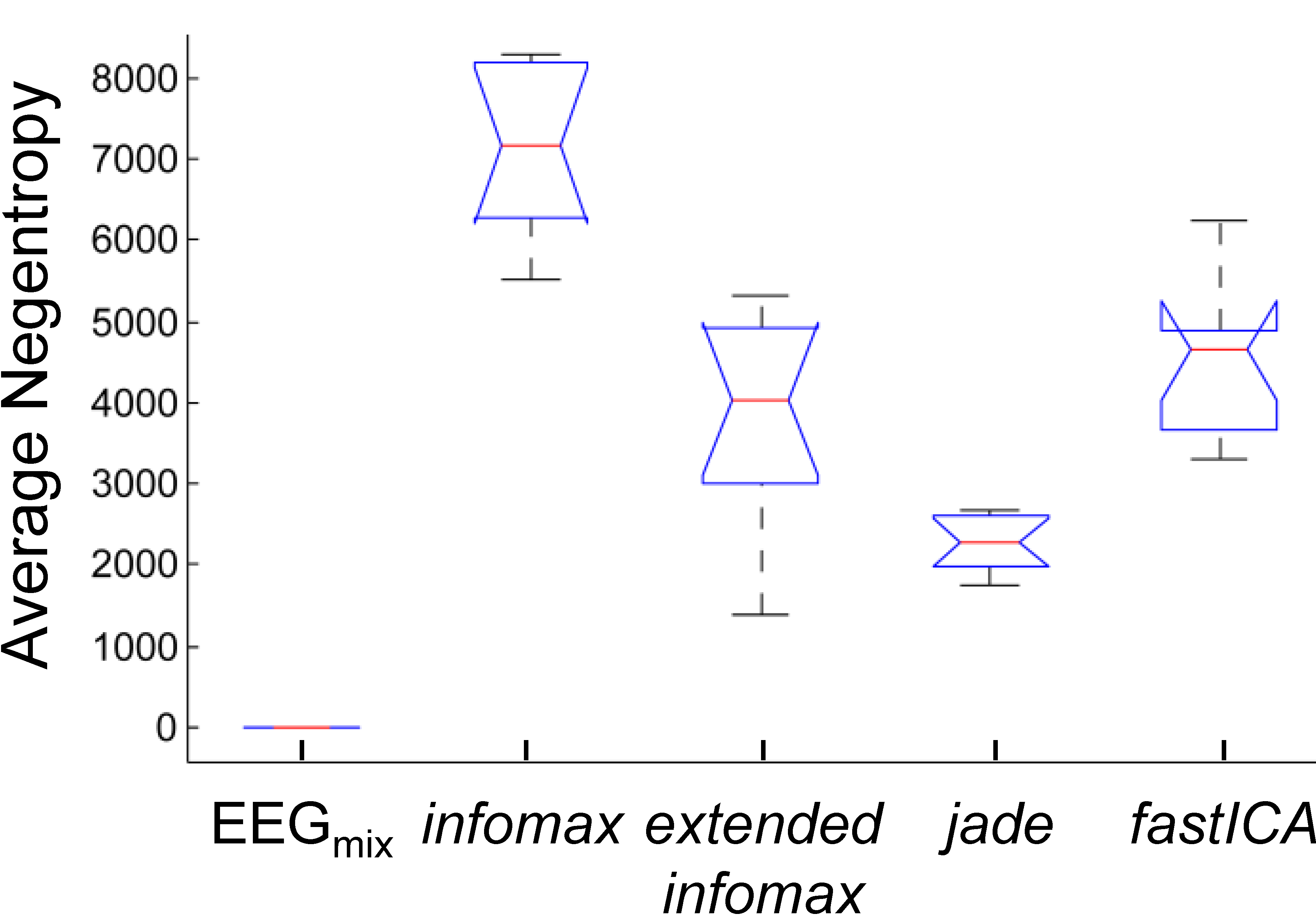

Supplement: S1 Fig — The depicted boxplots illustrate the average negentropy obtained for the channels of EEGmix as well as for the estimated sources by each of the ICA algorithms tested (n = 10 simulations). The mean values and their standard deviations are given in Table 2 of the manuscript. (TIF) [file pone.0174462.s015.tif]
